# Somatic Mutation Allelic Ratio Test Using ddPCR (SMART-ddPCR): An Accurate Method for Assessment of Preferential Allelic Imbalance in Tumor DNA

**DOI:** 10.1371/journal.pone.0143343

**Published:** 2015-11-17

**Authors:** Adam J. de Smith, Kyle M. Walsh, Helen M. Hansen, Alyson A. Endicott, John K. Wiencke, Catherine Metayer, Joseph L. Wiemels

**Affiliations:** 1 Department of Epidemiology and Biostatistics, University of California San Francisco, San Francisco, California, United States of America; 2 Division of Neuroepidemiology, Department of Neurological Surgery, University of California San Francisco, San Francisco, California, United States of America; 3 School of Public Health, University of California, Berkeley, California, United States of America; Ohio State University Medical Center, UNITED STATES

## Abstract

The extent to which heritable genetic variants can affect tumor development has yet to be fully elucidated. Tumor selection of single nucleotide polymorphism (SNP) risk alleles, a phenomenon called preferential allelic imbalance (PAI), has been demonstrated in some cancer types. We developed a novel application of digital PCR termed Somatic Mutation Allelic Ratio Test using Droplet Digital PCR (SMART-ddPCR) for accurate assessment of tumor PAI, and have applied this method to test the hypothesis that heritable SNPs associated with childhood acute lymphoblastic leukemia (ALL) may demonstrate tumor PAI. These SNPs are located at *CDKN2A* (rs3731217) and *IKZF1* (rs4132601), genes frequently lost in ALL, and at *CEBPE* (rs2239633), *ARID5B* (rs7089424), *PIP4K2A* (rs10764338), and *GATA3* (rs3824662), genes located on chromosomes gained in high-hyperdiploid ALL. We established thresholds of AI using constitutional DNA from SNP heterozygotes, and subsequently measured allelic copy number in tumor DNA from 19–142 heterozygote samples per SNP locus. We did not find significant tumor PAI at these loci, though *CDKN2A* and *IKZF1* SNPs showed a trend towards preferential selection of the risk allele (p = 0.17 and p = 0.23, respectively). Using a genomic copy number control ddPCR assay, we investigated somatic copy number alterations (SCNA) underlying AI at *CDKN2A* and *IKZF1*, revealing a complex range of alterations including homozygous and hemizygous deletions and copy-neutral loss of heterozygosity, with varying degrees of clonality. Copy number estimates from ddPCR showed high agreement with those from multiplex ligation-dependent probe amplification (MLPA) assays. We demonstrate that SMART-ddPCR is a highly accurate method for investigation of tumor PAI and for assessment of the somatic alterations underlying AI. Furthermore, analysis of publicly available data from The Cancer Genome Atlas identified 16 recurrent SCNA loci that contain heritable cancer risk SNPs associated with a matching tumor type, and which represent candidate PAI regions warranting further investigation.

## Introduction

Over the past decade, genome-wide association studies (GWAS) have identified common heritable genetic variants associated with most cancer types. Similarly, the advent of next generation sequencing has illuminated the landscape of acquired genetic variation in cancer, which includes point mutations and copy number alterations (reviewed in Vogelstein *et al*. 2013 [1]). However, the relationship between heritable and somatic genetic alterations during tumorigenesis is relatively underexplored.

Heritable risk variants have been shown to interact with somatic changes that occur during tumor development [2, 3]. For instance, tumor selection of cancer-associated single nucleotide polymorphism (SNP) risk alleles has been demonstrated in colorectal cancer [4–6] and squamous cell carcinoma [2, 7]. This type of selection, known as preferential allelic imbalance (PAI), may occur when there is somatic loss or gain at a genomic locus containing a heritable cancer-associated SNP, whereby the risk allele is preferentially retained or gained relative to the protective allele. Several techniques have previously been used to investigate tumor PAI, including Sanger sequencing [6], microsatellite marker genotyping [2, 5], and SNP genotyping [4, 7]. These methodologies do not allow absolute quantitation of allelic imbalance (AI), and also provide no indication as to the clonality of the specific SNP allele loss or gain within the tumor.

We describe in detail a novel method utilizing digital PCR technology to investigate PAI in tumor DNA, termed “SMART-ddPCR” (Somatic Mutation Allelic Ratio Test using Droplet Digital^TM^ PCR). We have applied this method to test the hypothesis that heritable SNPs associated with risk of acute lymphoblastic leukemia (ALL), the most common childhood cancer [8], may show PAI in the tumor. In a recent fine-mapping analysis of the ALL association signal at chromosome 9p21.3, we identified that the risk allele of the top hit SNP rs3731249, a missense variant in *CDKN2A*, was preferentially retained in subjects with somatic loss at this locus [9]. In the current study, we used SMART-ddPCR to test tumor PAI at five additional genomic loci associated with risk of childhood ALL, including at *IKZF1* (chr7p12.2), *CEBPE* (chr14q11.2), *ARID5B* (chr10q21.2), *PIP4K2A* (chr10p12.2), and *GATA3* (chr10p14) [10–14], in addition to the original GWAS tagging SNP at *CDKN2A*. Copy number at all 6 loci is often somatically altered in the leukemia, with deletions of *CDKN2A/B* and *IKZF1* occurring in ~30% and ~15% of cases respectively [15, 16], and with the remaining GWAS hits located on chromosomes 10 (*ARID5B*, *PIP4K2A*, and *GATA3*) and 14 (*CEBPE*), both of which are frequently gained in the high-hyperdiploid (HeH) subtype of ALL [17]. For SNPs at gene deletion loci we predicted that risk alleles would be preferentially retained in heterozygotes with somatic gene loss, and for SNPs on chromosomes gained in HeH ALL we predicted that risk alleles would be gained relative to protective alleles.

Through assessment of childhood ALL-associated SNPs, we demonstrate SMART-ddPCR to be a highly accurate and straightforward tool for investigating PAI in tumor DNA, as well as providing information on somatic copy number changes. In addition, we have mined publicly available datasets to identify cancer-associated SNPs located in regions of common somatic copy number alteration, thus revealing candidate regions for future PAI analyses.

## Materials and Methods

### Ethics Statement

This study was reviewed and approved by institutional review committees at the University of California Berkeley, the California Department of Public Health (CDPH), and all collaborating institutions. Written informed consent was obtained from all parent respondents.

### Study subjects

Study subjects were enrolled in the California Childhood Leukemia Study (CCLS) as previously described [18]. Briefly, the CCLS is a continuing case-control study initiated in 1995, and includes childhood ALL cases recruited from 35 counties in Northern and Central California. Information from birth certificates obtained from the California Office of Vital Records was used to select one or two controls for each case, matching on age (birthdate), sex, Hispanic ethnicity, and maternal race. For subjects to be eligible, they had to reside in the study area, be younger than 15 years of age at diagnosis (reference date for matched controls), have at least one English or Spanish-speaking parent/guardian, and have no history of cancer diagnosis. Approximately 85% of eligible cases and 86% of contacted eligible controls consented to participate. In this manuscript, we describe data generated from cases only.

For SNP genotyping, 297 Hispanic children with B-cell ALL (B-ALL) and 24 children with T-cell ALL (T-ALL) were included from the CCLS. Constitutional DNA from neonatal bloodspots was genotyped using Illumina OmniExpress genome-wide SNP arrays, as previously described [19]. For the *CDKN2A* SNPs investigated for tumor PAI, we included both B-ALL and T-ALL subjects, as rs3731217 is a risk locus for both subtypes. For SNPs in other genes, we included only B-ALL subjects (n = 297). The genotype information available for the leukemia-associated SNPs that are the focus of this study is summarized in Table 1. Genotype data for additional subjects was available for SNPs in *CDKN2A*, *IKZF1*, *CEBPE*, and *ARID5B* from replication analyses carried out by either Taqman or Sequenom genotyping assays, as previously described [9, 20] (Table 1).

**Table 1:** Summary of the childhood ALL-associated SNPs investigated and the corresponding tumor DNA allelic imbalance results.

Gene	Tumor-associated SCNA (predicted %)	SNP	Genomic location (hg19)	Genotyped ALL cases *n* (GWAS/ replication)	Tumor heterozygote samples *n* *	Samples with AI *n* (%)	Risk allele PAI *n*	Protective allele PAI *n*	p-value (1-sided)
*CDKN2A*	Deletion (~28%)	rs3731217	chr9:21984661	653 (321/332)	50	17 (34.0)	11	6	0.17
*IKZF1*	Deletion (~15%)	rs4132601	chr7:50470604	543 (297/246)	142	29 (20.4)	17	12	0.23
*CEBPE*	Gain of chr14 (~91%)‡	rs2239633	chr14:23589057	570 (297/273)	42†	32 (76.2)	19	13	0.19
*ARID5B*	Gain of chr10 (~67–76%)‡	rs7089424	chr10:63752159	543 (297/246)	61†	35 (57.4)	20	15	0.25
*PIP4K2A*	Gain of chr10 (~67–76%)‡	rs10764338	chr10:22866892	297 (297/0)	19†	9 (47.4)	4	5	0.5
*GATA3*	Gain of chr10 (~67–76%)‡	rs3824662	chr10:8104208	297 (297/0)	37†	19 (51.4)	10	9	0.5

* Number of heterozygous samples (for each SNP) with available bone marrow (i.e. tumor) DNA.

‡ % of HeH ALL samples with gains of that chromosome, based on data from Paulsson *et al*. (2010) [21] and Dastugue *et al*. (2013) [22].

† High hyperdiploid samples only.

Significant p-values highlighted in bold.

### SNP and sample selection

Our recent fine-mapping analysis at the *CDKN2A* region revealed a missense SNP rs3731249 that confers ~3-fold risk of ALL [9]. Using SMART-ddPCR, we found that the rs3731249 risk allele showed significant tumor PAI: of 17 tumor samples from SNP heterozygotes, 14 had AI favoring the risk allele versus only 3 with AI favoring the protective allele (p = 0.006, 1-sided binomial significance test). In the current study, we wished to determine whether the original tagging *CDKN2A* SNP rs3731217, identified by Sherborne *et al*. (2010) [12], also shows tumor PAI. Another significant risk factor is *IKZF1*, a locus that also incorporates ALL-associated SNPs and is commonly deleted in the tumor. We predicted, therefore, that *IKZF1* SNP risk alleles may be preferentially retained when hemizygous *IKZF1* loss occurs in the tumor. The original GWAS top hit SNP rs4132601 was selected to test this hypothesis [11].

SNPs in *ARID5B*, *CEBPE*, and *PIP4K2A* are more strongly associated with HeH B-ALL [11, 13, 23] (Table 1). As these SNPs are located on chromosomes frequently gained in HeH B-ALL, we predicted that their risk alleles may be preferentially gained relative to protective alleles. For *ARID5B* and *CEBPE*, we selected the original GWAS top hit SNPs, rs7089424 and rs2239633 respectively [11]. For *PIP4K2A*, we selected the top directly genotyped SNP from our dataset–rs10764338 –which was exclusively associated with HeH B-ALL [23].

A recent GWAS identified SNP rs3824662 in *GATA3* to be specifically associated with the Philadelphia chromosome-like (Ph-like) subtype of ALL [24]. Somatic alterations at the *GATA3* locus are not associated with this subtype. Moreover, rs3824662 was not associated with HeH ALL, thus we predicted that the risk allele of this variant would not be preferentially gained in heterozygote HeH subjects.

For each of the ALL risk SNPs, we identified all heterozygous ALL patients with available diagnostic (pre-treatment) bone marrow (*i*.*e*. tumor) DNA (Table 1). For SNPs more strongly associated with HeH B-ALL and/or located on chromosomes frequently gained in HeH B-ALL (*CEBPE*, *ARID5B*, *PIP4K2A*, and *GATA3*), we limited subjects to those identified as having this ALL subtype.

### DNA extraction

DNA was extracted from leukemia diagnostic bone marrow (*i*.*e*. tumor) samples using the QIAamp DNA Mini Kit (QIAGEN). Constitutional DNA was extracted for cases from neonatal bloodspots using the QIAamp DNA Micro Kit (QIAGEN). DNA sample concentrations ranged from 1ng/μl to 20ng/μl.

### Somatic Mutation Allelic Ratio Test using Droplet Digital PCR (SMART-ddPCR)

#### i). Assessing allelic imbalance of leukemia-associated SNPs in tumor DNA

To assess preferential allelic imbalance (PAI) of the childhood ALL-associated SNPs in tumor DNA, the QX100™ Droplet Digital™ PCR System (Bio-Rad) was used to determine copy number of the risk and protective alleles in tumor DNA from known SNP heterozygotes. We termed this methodology Somatic Mutation Allelic Ratio Test using Droplet Digital^TM^ PCR, or “SMART-ddPCR”. ddPCR enables an absolute measure of DNA concentration, through partitioning of PCR reactions into 10–20,000 individual reactions as water-in-oil droplets, which are read through a droplet flow cytometer. For each SNP, a validated Taqman SNP Genotyping Assay (Applied Biosystems) was purchased, with FAM- and VIC-labeled probes for detection of the risk and protective alleles respectively. Therefore, the copy number of risk and protective alleles could be assessed in the same well.

ddPCR was carried out as previously described [25]. In brief, a ddPCR mastermix was made containing 11μl 2X ddPCR^TM^ Supermix (Bio-Rad), 1.1μl 20X Taqman SNP Genotyping Assay (Applied Biosystems), and 7.9μl nuclease-free H_2_0 (Qiagen) per sample. The mastermix was prepared at room temperature and 20μl was added to 2μl of each DNA sample. Samples were loaded into individual wells of DG8^TM^ cartridges (BioRad), and droplets were generated using a QX100 Droplet Generator (BioRad). For each sample, 40μl of droplet mix was then transferred to a 96-well plate, and PCR was performed in a thermal cycler using the following cycling conditions: 95°C x 10 min; 40 cycles of [94°C x 30s, 60°C x 60s]; 98°C x 10s; 40C x 10min. The Bio-Rad QX100^TM^ Droplet Reader was then used to assess droplets as positive or negative based on fluorescence amplitude. The QuantaSoft software (BioRad) was used to analyze droplet data.

For each SNP assay, heterozygote samples with available tumor DNA were run in duplicate. To determine presence of AI, the proportion of risk allele relative to protective allele was calculated for each subject using the following equation:

$$Risk\;allele\;proportion=\frac{Mean\;conc.\;\left(\frac{copies}{\mu L}\right)of\;SNP\;risk\;allele}{\left(Mean\;conc.\;of\;SNP\;risk\;allele+mean\;conc.\;of\;SNP\;protective\;allele\right)}$$

The resulting risk allele proportion lies between 0 and 1, with an expected proportion of 0.50 for samples without AI (*i*.*e*. mean conc. of risk allele = mean conc. of protective allele). Samples with low concentrations for both risk and protective alleles were excluded.

For each SNP, we determined upper and lower thresholds of AI by carrying out repeat measurements on constitutional DNA from heterozygote cases, which should have a risk allele proportion of 0.5 due to lack of copy number alterations in the germline. Constitutional DNA was not available for every case, therefore upper and lower thresholds were defined by 3 standard deviations above and below the mean risk allele proportion in these samples, according to the three-sigma rule. Tumor samples with risk allele proportions above or below the upper/lower thresholds were defined as having AI (S1 Table).

#### ii). Assessing somatic copy number

For SNPs located in genomic loci frequently deleted in ALL, *i*.*e*. *CDKN2A* and *IKZF1*, we investigated whether AI was caused by somatic copy number loss or by copy-neutral loss of heterozygosity (LOH). In addition, we wished to assess clonality of these alterations. Thus, a second ddPCR reaction was carried out using a Taqman assay targeting the *SLC24A3* gene within a region not known to vary in copy number in ALL [26]. For *CDKN2A*, somatic copy number was assessed in 35 samples heterozygous for the missense SNP rs3731249. For *IKZF1*, copy number was assessed in 75 of the 142 rs4132601 heterozygote samples with sufficient DNA remaining. The total concentration (*i*.*e*. copy number) of each gene relative to concentration of the genomic control was calculated as follows:

$$Test\;gene\;concentration=\frac{\left(Mean\;conc.\;of\;SNP\;risk\;allele+mean\;conc.\;of\;SNP\;protective\;allele\right)}{Mean\;genomic\;control\;conc.\;\left(SLC24A3\right)}$$

Tumor samples that presented with copy number loss but without AI were presumed to have subclonal homozygous deletions. Conversely, samples with no evidence of copy-number loss but showing evidence of AI were presumed to have copy-neutral LOH.

### Multiplex ligation-dependent probe amplification

To assess the accuracy of the ddPCR copy number estimates, an alternate method was used to measure somatic *CDKN2A* and *IKZF1* copy number. Multiplex ligation-dependent probe amplification (MLPA) was carried out using the SALSA MLPA probemix P335-B1 ALL-IKZF1 (MRC Holland, The Netherlands), which includes 3 probes overlapping the *CDKN2A*/*B* locus, and 8 probes across *IKZF1* (one probe within each exon) [16]. MLPA was performed as previously described [27] for subjects with SMART-ddPCR data and sufficient bone marrow DNA available (27 samples for *CDKN2A* and 75 samples for *IKZF1*). Analysis of MLPA data was carried out using the “Coffalyser.Net” fragment analysis software (MRC Holland). In brief, peak height ratios were determined by intra-sample normalization using data from 13 reference probes in genomic regions not known to be somatically altered in childhood ALL, and by inter-sample normalization using data from control (constitutional) DNA subjects.

### PCR and Sanger sequencing

To assess SMART-ddPCR measurements of AI using an alternative method, we carried out PCR and Sanger sequencing for one deletion locus, *IKZF1* SNP rs4132601, and one copy number gain locus, *ARID5B* SNP rs7089424. PCR primers were designed using the Primer3 software (http://primer3.ut.ee) and PCR reactions carried out using the Advantage 2 PCR kit (Clontech) for 6 constitutional and 13 tumor DNA samples heterozygous for rs4132601, and for 7 constitutional and 17 tumor samples heterozygous for rs7089424. PCR products were cleaned up using ExoSAP-IT reagent (Affymetrix), and sequenced bi-directionally using an ABI 3730xl DNA sequencer. Sequence chromatogram files were analyzed using Chromas software (Technelysium). For each sample, SNP risk allele proportions were calculated for both forward and reverse sequences as follows:

$$Risk\;allele\;proportion=\frac{Peak\;height\;of\;SNP\;risk\;allele}{\left(Peak\;height\;of\;SNP\;risk\;allele+peak\;height\;of\;SNP\;protective\;allele\right)}$$

The mean risk allele proportion across forward and reverse sequences was then determined. As described above for SMART-ddPCR, thresholds of AI were calculated from repeat measurements on constitutional DNA from a subset of heterozygote cases.

### Statistical analysis

For each SNP, a binomial significance test was used to determine whether the number of samples with PAI favoring the risk allele was significantly different than the number of samples with PAI favoring the protective allele. We assumed, under the null hypothesis, that a sample was equally likely to lose one allele as the other (*i*.*e*. p = q = 0.50), though we used a one-sided binomial test as we had *a priori* predictions of the direction of AI for each SNP.

The “Agreement” package was used in *R* to calculate the concordance correlation coefficient (CCC) [28] between copy number measurements made by the ddPCR and MLPA assays for both *CDKN2A* and *IKZF1*. The CCC statistic combines measures of both precision and accuracy to determine the extent to which observed data deviate from the line of perfect concordance. Bland-Altman plots were generated using the “MethComp” *R* package.

### *In silico* prediction of cancer-associated SNPs that may show tumor PAI

To identify candidate SNPs that might show tumor PAI in other cancer types, an integrated *in silico* analysis of reported recurrent somatic copy number alterations (SCNAs) and cancer-associated SNPs was carried out. Pan-cancer regions of significant SCNAs were retrieved from The Cancer Genome Atlas (TCGA) Pan-Cancer dataset [29], which was based on analysis of 4,934 tumor samples across 11 different cancer types. This dataset included 140 significantly recurrent SCNAs– 70 recurrently amplified and 70 recurrently deleted–with peak regions identified that most likely contain oncogenes or tumor suppressor genes targeted by these SCNAs. The list of 140 genomic loci was compared with the latest National Human Genome Research Institute (NHGRI) GWAS Catalog [30] to identify cancer-associated SNPs that overlapped the chromosomal locations of these SCNAs. For each SCNA, GWAS SNPs were included if their chromosomal position overlapped the SCNA ‘peak’ region and/or if they overlapped gene(s) listed in the peak region [29] (S2 and S3 Tables). We then determined SCNA regions at which the type of cancer associated with the overlapping GWAS SNPs matched the type of cancer in which the amplification or deletion was identified in TCGA (*i*.*e*. a heritable SNP associated with cancer “X” lies within a region commonly deleted/amplified in tumor tissue from cancer “X”).

## Results

### SMART-ddPCR analysis of tumor PAI at childhood ALL SNP loci

For all 6 SNP assays, repeat ddPCR measurements of constitutional DNA from SNP heterozygotes resulted in risk allele proportions at or very close to the expected proportion of 0.5, with a mean risk allele proportion across the 7 SNPs of 0.5 (range: 0.488–0.512). The mean upper and lower AI thresholds, calculated as defined in Methods, were 0.548 and 0.453 respectively (S1 Table).

**Fig 1:**
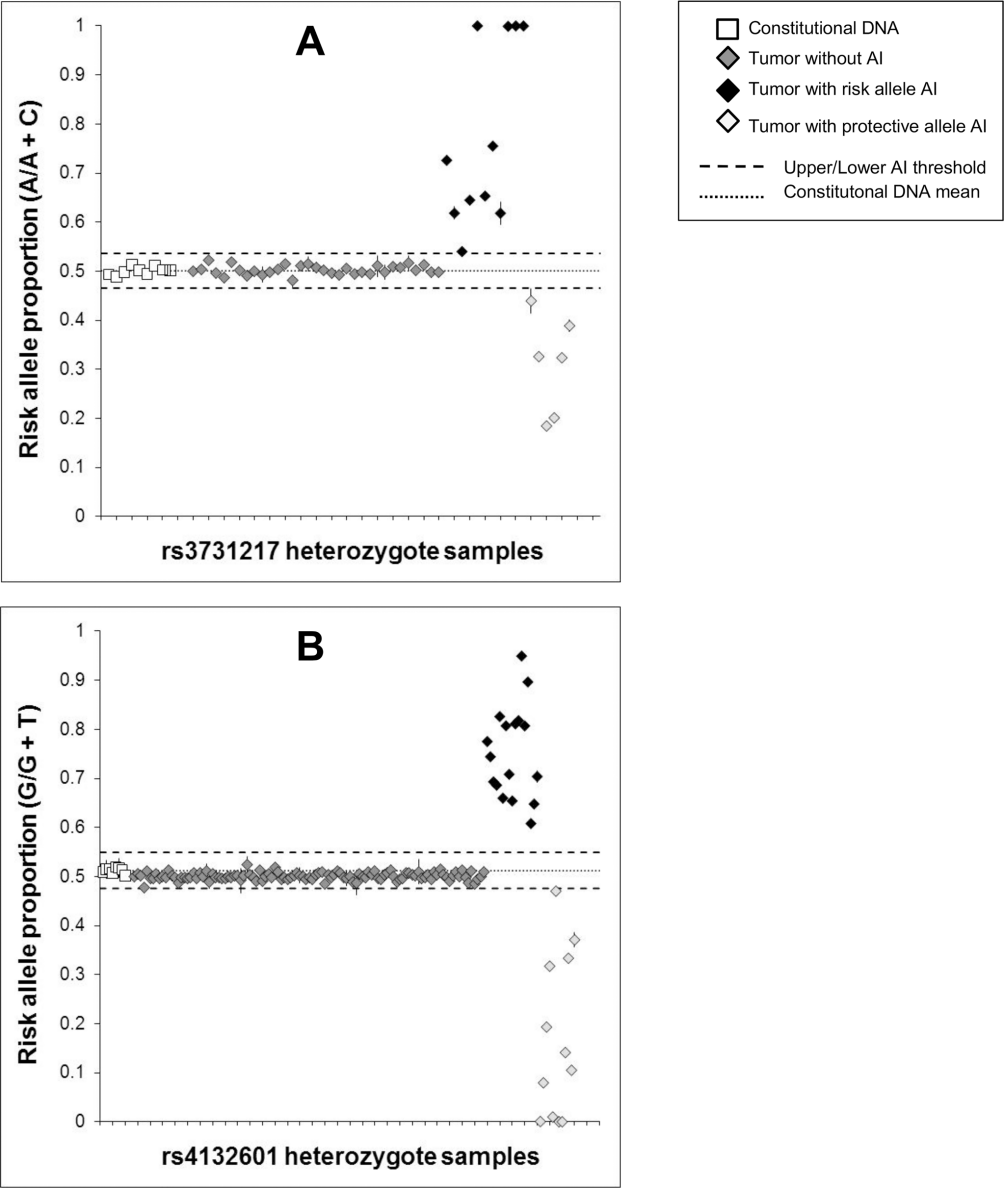
Risk allele proportions at genomic loci with somatic loss. Allelic copy number was measured in constitutional DNA and leukemia bone marrow (tumor) DNA from ALL patients heterozygous for *CDKN2A* tagging SNP rs3731217 (A), and *IKZF1* SNP rs4132601 (B). Risk allele proportions are displayed as a fraction of the total allelic copy number measured in each patient using ddPCR. Each subject was assayed in duplicate, and error bars represent the standard error of the mean (some error bars not visible due to their range falling within boundaries of the data point). Upper/lower thresholds of allelic imbalance (AI) were +/- 3 SDs from the mean allelic proportion from repeat measurements in constitutional DNA samples (white squares). For rs3731217, 11 tumor samples showed AI favoring the risk allele versus 6 patients with AI favoring the protective allele (P = 0.17). For rs4132601, 17 tumor samples showed AI favoring the risk allele versus 12 patients with AI favoring the protective allele (P = 0.23).

For SNPs in *CDKN2A* and *IKZF1*, genes frequently deleted in ALL, we hypothesized *a priori* that the risk allele would be preferentially retained when somatic loss occurs at these loci in heterozygote cases. We previously reported significant tumor risk allele PAI for the *CDKN2A* missense SNP rs3731249 [9]. Although not significant, the tagging SNP rs3731217 showed a trend in the same direction. Out of 50 heterozygote tumor samples, 17 (34.0%) showed evidence of AI, of which 11 demonstrated preferential retention of the risk allele versus 6 with retention of the protective allele (p = 0.17) (Table 1, Fig 1). For the *IKZF1* SNP rs4132601, 29 out of 142 (20.4%) samples showed AI, of which 17 showed preferential retention of the risk allele versus 12 with retention of the protective allele (p = 0.23) (Table 1, Fig 1). Though not significant, this was again in the predicted direction of tumor PAI favoring the risk allele. For both the *CDKN2A* and *IKZF1* SNPs, it was interesting to note a range of risk allele proportion values above and below the AI thresholds (Fig 1). We would expect constitutionally heterozygous samples with no somatic gene loss to maintain a risk allele proportion of 0.5, whereas samples in which a hemizygous deletion has occurred early in clonal evolution should have a risk allele proportion at approximately 1.0 or 0, with either the protective or risk allele lost in all clones expanding in that individual. Risk allele proportions deviating from 0.5, 1.0 or 0 likely represent samples with subclonal gene loss that formed later in clonal evolution, highlighting that patient tumor DNA includes a population of tumor cells that may represent a range of expanded clones.

**Fig 2:**
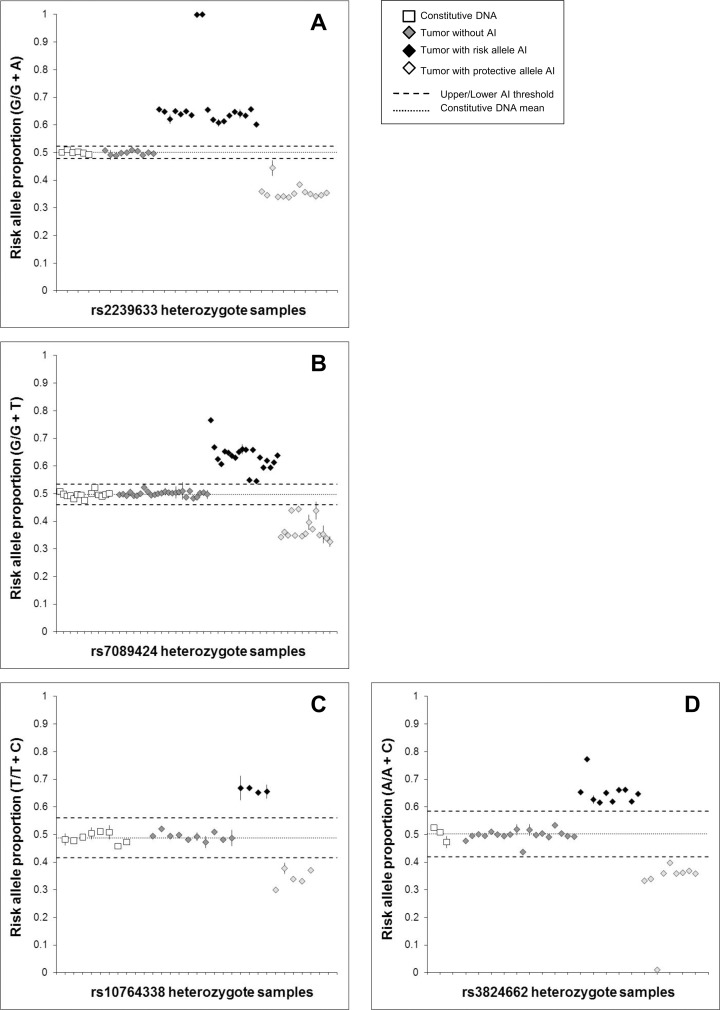
Risk allele proportions at genomic loci with somatic gain (*i*.*e*. hyperdiploid chromosomes). Allelic copy number was measured in constitutional DNA and leukemia bone marrow (tumor) DNA from HeH ALL patients heterozygous for ALL-associated SNPs on chromosomes frequently gained in HeH ALL: *CEBPE* SNP rs2239633 (A), *ARID5B* SNP rs7089424 (B), *PIP4K2A* SNP rs10764338 (C), and *GATA3* SNP rs3824662 (D). Risk allele proportions are displayed as a fraction of the total allelic copy number measured in each patient using ddPCR. Each subject was assayed in duplicate, and error bars represent the standard error of the mean (some error bars not visible due to their range falling within boundaries of the data point). Upper/lower thresholds of allelic imbalance (AI) were +/- 3 SDs from the mean allelic proportion from repeat measurements in constitutional DNA samples (white squares). For rs2239633, 19 tumor samples showed AI favoring the risk allele versus 13 patients with AI favoring the protective allele (P = 0.19). For rs7089424, 20 tumor samples showed AI favoring the risk allele versus 15 patients with AI favoring the protective allele (P = 0.25). For rs10764338, 4 tumor samples showed AI favoring the risk allele versus 5 patients with AI favoring the protective allele (P = 0.50). For rs3824662, 10 tumor samples showed AI favoring the risk allele versus 9 patients with AI favoring the protective allele (P = 0.50). Data points clustering at ~0.66 and ~0.33 represent a 3:2 or 2:3 risk:protective allele proportion due to chromosomal copy number shifting from diploid (n = 2) to triploid (n = 3). Data points at ~0.75 represents a 3:1 risk:protective allele proportion due to a diploid to tetraploid (n = 4) shift in chromosome ploidy. Data points at 1 and 0 likely represent HeH ALL that has arisen via near-haploidy, leading to chromosomal LOH (Paulsson *et al*. 2005) [31].

For SNPs in *CEBPE*, *ARID5B*, and *PIP4K2A*, which are associated with HeH ALL and which are located on chromosomes frequently gained in this subtype, we hypothesized that risk alleles would be preferentially gained in heterozygote HeH cases. Both the *ARID5B* and *CEBPE* SNPs showed a trend towards preferential gain of the risk allele, though neither result was significant (Table 1, Fig 2). For *PIP4K2A*, only 9 samples showed evidence of AI, and there was no evidence of risk allele PAI (p = 0.5). For *GATA3*, we predicted that the SNP risk allele would not show tumor PAI in HeH ALL subjects, and this was the case (p = 0.5).

For *CEBPE*, 76.2% tumor samples showed evidence of AI, compared with a mean of 52.1% for the chromosome 10 SNPs, reflecting that chromosome 14 is more frequently gained in HeH ALL than chromosome 10 [21, 22]. Risk allele proportions for the chromosome 10 and 14 SNPs were much more tightly clustered than for the deletion genes *CDKN2A* and *IKZF1* (Figs 1 and 2). Data points at ~0.67 and ~0.33 represent a 3:2 or 2:3 risk:protective allele ratio due to chromosomal copy number shifting from diploid (n = 2) to triploid (n = 3). Data points at ~0.75 represents a 3:1 risk:protective ratio proportion due to a diploid to tetraploid (n = 4) shift. Data points at 1 (complete loss of protective allele) and 0 (loss of risk allele) likely represent HeH ALL that has arisen via near-haploidy leading to chromosomal LOH, which is a less frequent origin of formation for this leukemia subtype [31]. The small number of samples that did not cluster at these values may represent a mixture of healthy B-cells in the leukemia bone marrow sample, or simply noisy samples that do not represent genuine AI.

To validate SMART-ddPCR measurements of AI using a second method, we carried out Sanger sequencing across the *IKZF1* and *ARID5B* SNPs for constitutional and tumor DNA from SNP heterozygotes. There was a very high correlation between tumor sample risk allele proportions measured by SMART-ddPCR and by Sanger sequencing for both rs4132601 (R^2^ = 0.98) and rs7089424 (R^2^ = 0.97), as shown in S1 and S2 Figs. The majority of tumor samples that demonstrated AI by SMART-ddPCR also showed evidence of AI by Sanger sequencing (8/9 for rs4132601 and 11/12 for rs7089424). However, there were 2 subjects in which AI could not be validated, perhaps due to the reduced sensitivity of Sanger sequencing (S1 and S2 Figs). Furthermore, for both SNPs the risk allele proportions were shifted towards increased risk allele, with a mean risk allele proportion across repeat measures of constitutional DNA of 0.574 (range: 0.565–0.589) and 0.527 (range: 0.505–0.544) for rs4132601 and rs7089424 respectively, demonstrating the reduced accuracy of Sanger sequencing compared with SMART-ddPCR (S1 and S2 Figs).

### Analysis of somatic copy number at *CDKN2A* and *IKZF1*

We explored the clonality of alterations at *CDKN2A* and *IKZF1* by carrying out ddPCR with a genomic control assay at *SLC24A3*, a locus known not to vary in copy number in ALL. By normalizing to this control locus, we determined genomic copy number estimates of *CDKN2A* and *IKZF1* in tumor DNA samples.

**Fig 3:**
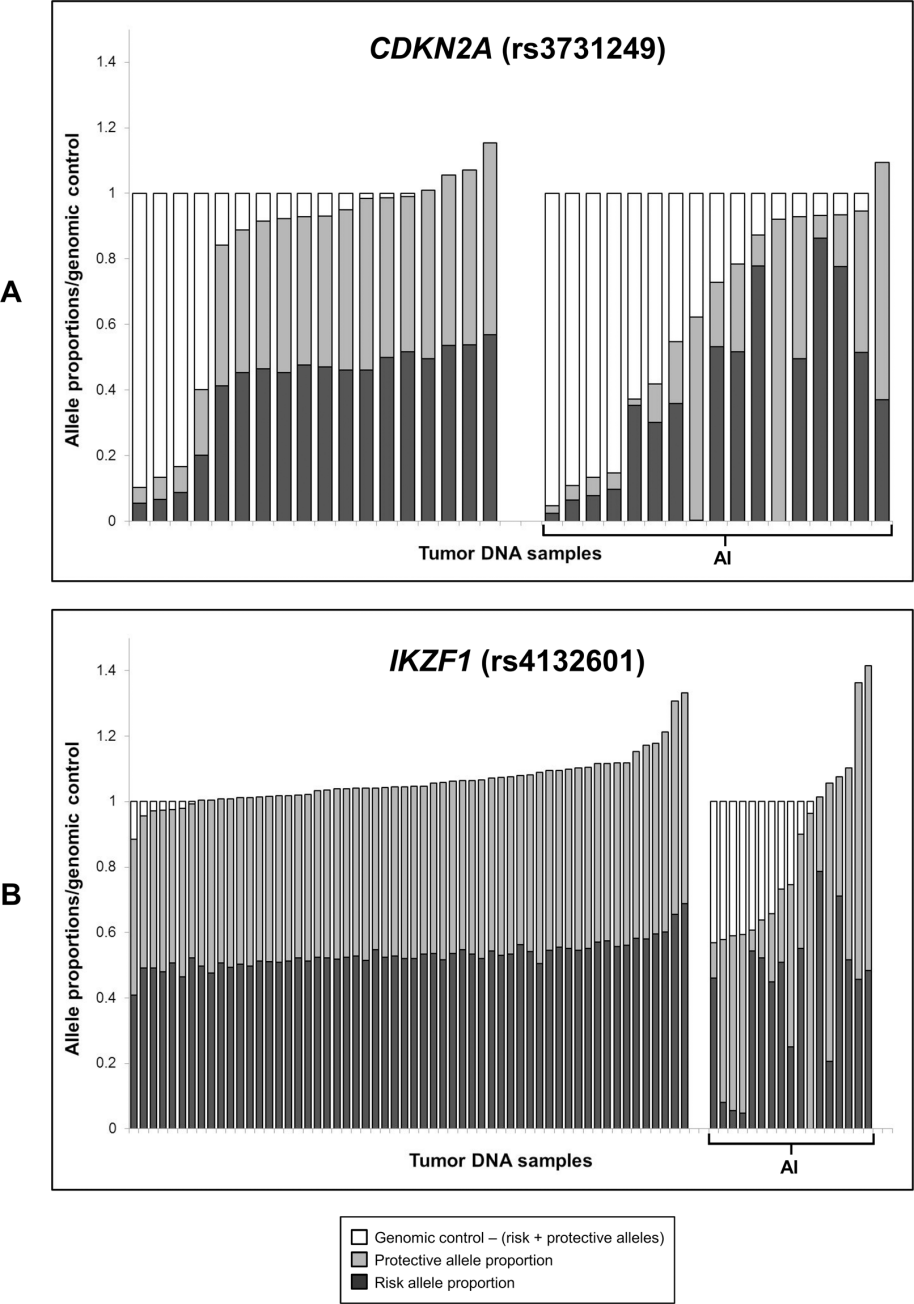
*CDKN2A* and *IKZF1* SNP allele proportions in tumor DNA relative to genomic control copy number. Stacked histograms showing tumor DNA copy number of (A) *CDKN2A* and (B) *IKZF1* SNPs relative to a genomic control locus (*SLC24A3*). Black and grey bars represent the proportions of normalized SNP copy number accounted for by the risk and protective alleles respectively. White bars represent the difference between *CDKN2A*/*IKZF1* SNP copy number and the genomic control gene copy number. SMART-ddPCR was used to measure copy number of SNP risk/protective alleles, as well as the genomic control locus, in 35 leukemia bone marrow (tumor) DNA samples for *CDKN2A* (SNP rs3731249) and 75 tumor DNA samples for *IKZF1* (SNP rs4132601). Samples are grouped into those with allelic imbalance (AI) and those without AI, and arranged in order of normalized gene copy number relative to the genomic control.

For *CDKN2A*, a complex range of somatic alterations was apparent (Fig 3). Of the 18 tumor samples that did not show AI, at least 4 samples presented with clear copy number loss and, thus, were presumed to have acquired subclonal homozygous deletions. Of the 17 samples that did show AI, there appeared to be an assortment of subclonal homozygous and hemizygous deletions (or a combination of homozygous loss with copy-neutral LOH), resulting in normalized *CDKN2A* copy number values ranging from 0.05 to 0.87. In addition, several samples had AI but no or little copy number loss, indicating copy-neutral LOH. In some of these samples, a small amount of the alternate allele was still present, suggesting subclonal copy-neutral LOH, or possible contamination of leukemia cells with non-leukemic cells.

In the 75 samples tested for *IKZF1*, there was no evidence of subclonal homozygous deletions in either the 58 samples without AI or the 17 samples with AI (Fig 3). Several samples with AI appeared to have clonal hemizygous deletions with loss of either the risk or protective allele, whilst other samples appeared to have subclonal hemizygous *IKZF1* loss. There was also evidence of both clonal and subclonal copy-neutral LOH.

**Fig 4:**
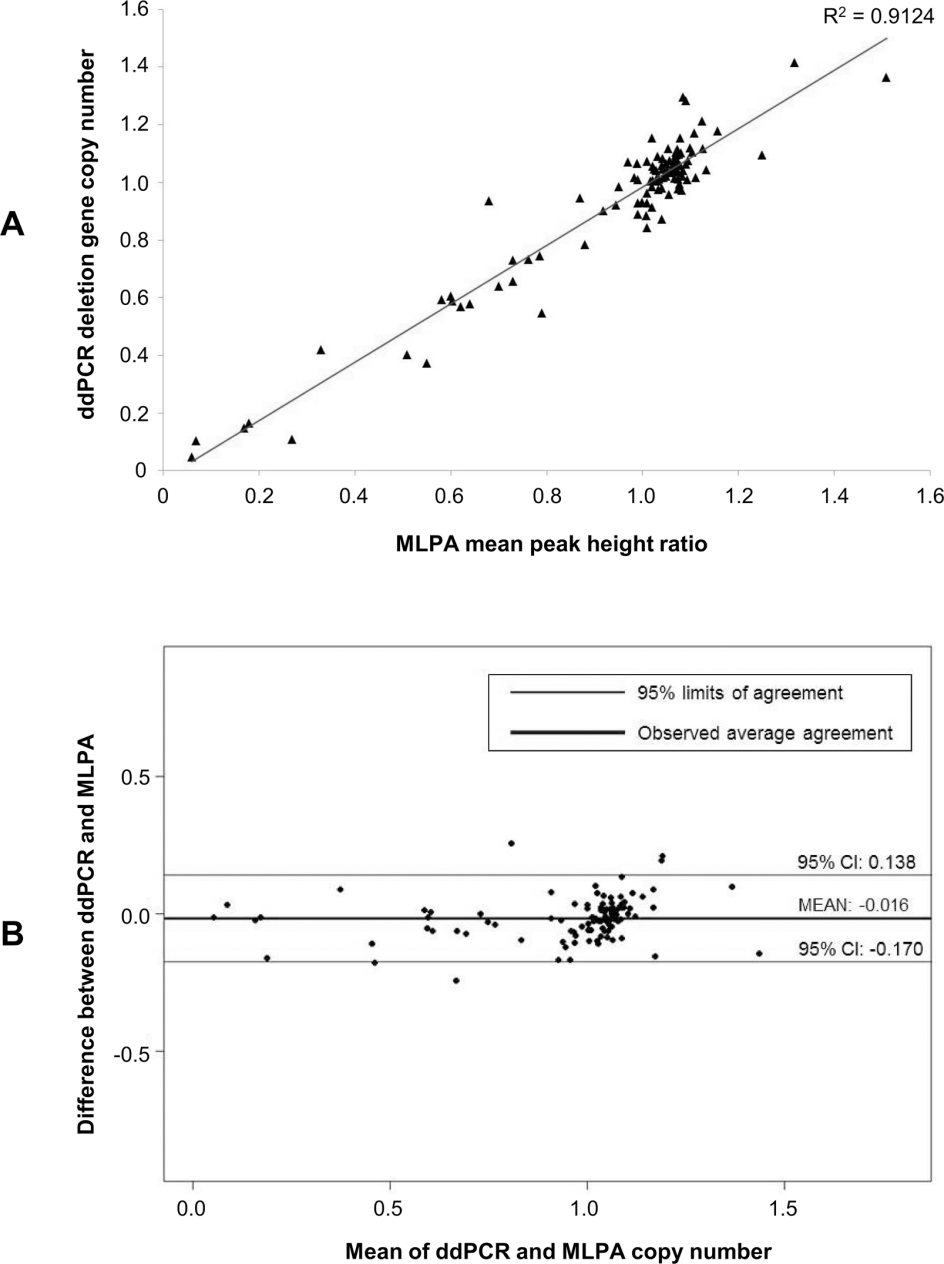
Comparison between deletion gene copy number measurements made by SMART-ddPCR and MLPA. Copy number measurements were available from ddPCR and MLPA assays for SNPs at the two deletion genes *CDKN2A* (SNP rs3731249) and *IKZF1* (SNP rs4132601) in 27 and 75 tumor DNA samples respectively. (A) High correlation (R2 = 0.91) between the combined deletion gene copy number measurements made by ddPCR and MLPA. (B) Bland-Altman plot displaying the difference between measurements made in the same individual against their mean, as measured by two different methodologies (*i*.*e*. ddPCR and MLPA). There is very close agreement between the copy number measurements made by the two assays, as demonstrated by the narrow limits of agreement (-0.170 to 0.138) either side of the observed average agreement (-0.016).

We compared copy number estimates for both *CDKN2A* and *IKZF1* as measured by ddPCR and MLPA. The mean MLPA probe peak height ratio across the 102 samples tested was highly correlated with normalized copy number from the SMART-ddPCR data (R^2^ = 0.91) (Fig 4). We calculated the concordance correlation coefficient (CCC) between the two assays to be 0.95 (95% CI: 0.94–0.99). Our data, therefore, demonstrate a very high level of agreement between copy number measurements from ddPCR and those from MLPA analyses (Fig 4).

### Candidate regions of tumor PAI across multiple cancer types

We analyzed the overlap between recurrent SCNAs identified in TCGA [29] and cancer-associated SNPs reported in the NHGRI GWAS catalog to identify heritable genetic variants with the potential to undergo selection during tumor development. The latest iteration of the GWAS catalog (January 2015) [30] contained 19,469 SNP loci, of which 1220 SNPs were associated with any type of cancer. Of these 1220 cancer-associated SNPs, 195 SNPs overlapped the TCGA recurrent SCNA loci (S4 Table). Of the TCGA recurrent SCNA loci, there were 23/70 (32.9%) somatic amplification loci and 24/70 (34.3%) somatic deletion loci that overlapped cancer-associated SNPs (S2 and S3 Tables). Of these, there were 8 amplification loci and 8 deletion loci in which the associations of overlapping SNPs matched the cancer type in which the SCNA was found to be recurrent in TCGA (Table 2). Bladder cancer had the most loci of potential tumor PAI, with 6 regions where recurrent SCNAs overlapped bladder cancer-associated SNPs. Breast cancer was the second most frequent (n = 4), followed by glioma and lung cancer (n = 3), colorectal and endometrial cancer (n = 2), and ovarian cancer and kidney renal cell carcinoma (n = 1).

**Table 2:** Candidates for tumor PAI: recurrent SCNA loci from TCGA that overlap cancer-associated SNPs (NHGRI GWAS Catalog) identified in matching tumor types.

SCNA type	TCGA Peak Name	Cytoband	Chromosome*	Start	End	Size (bp)	Heritable SNP association cancer type**	SCNA tumor types ‡
**Amplifications**	*TERC*	3q26.2	3	169389459	169490555	101096	**Bladder**; glioma; melanoma; multiple myeloma	**BLCA**, LUAD, LUSC, UCEC
** **	*FGFR3*	4p16.3	4	1778797	1817427	38630	**Bladder**	**BLCA**, GBM, OV, UCEC
** **	*TERT*	5p15.33	5	1287704	1300024	12320	Basal cell carcinoma; **bladder**; breast; **lung adenocarcinoma**; **glioma**; melanoma; prostate; testicular germ cell	**BLCA**, CRC, **GBM**, HNSC, **LUAD**, LUSC, OV, UCEC
** **	*EGFR*	7p11.2	7	55075808	55093954	18146	**Glioma**	BLCA, BRCA, **GBM**, HNSC, LUAD, LUSC
** **	*MYC*	8q24.21	8	128739772	128762863	23091	**Bladder**; **breast**; CLL; CRC; **ovarian**; prostate; **renal cell carcinoma**	**BLCA**, **BRCA**, GBM, **KIRC**, **OV**, UCEC
** **	*CCND1*	11q13.3	11	69464719	69502928	38209	**Breast**; multiple myeloma; renal cell carcinoma	**BRCA**, LUAD
** **	*KDM5A*	12p13.33	12	1	980639	980638	**CRC**	BRCA, **CRC**, HNSC, LUAD, LUSC, OV
** **	*CCNE1*	19q12	19	30306758	30316875	10117	**Bladder**	**BLCA**, BRCA, GBM, LUSC, OV, UCEC
**Deletions**	*IKZF2*	2q34	2	211542637	214143899	2601262	Breast; **non-small cell lung cancer**	BLCA, **LUSC**, UCEC
** **	*LINC00290*	4q34.3	4	178911874	183060693	4148819	Multiple myeloma; **non-small cell lung cancer**	BLCA, GBM, KIRC, **LUAD**, **LUSC**, OV, UCEC
** **	5q15	5q15	5	73236070	114508587	41272517	Breast; **CRC**; GBM; gallbladder; Hodgkin's lymphoma; prostate; Wilms tumor	**CRC**, LUAD, LUSC
** **	*CDKN2A*	9p21.3	9	21865498	22448737	583239	ALL; basal cell carcinoma; **breast**; CLL; **glioma**; nasopharyngeal carcinoma; melanoma	BLCA, **BRCA**, CRC, **GBM**, HNSC, KIRC, LUAD, LUSC
** **	9p21.2	9p21.2	9	27572512	28982153	1409641	**Endometrial**	**UCEC**
** **	*RAD51B*	14q24.1	14	68275375	69288431	1013056	**Breast**; prostate	**BRCA**, CRC, OV
** **	15q12	15q12	15	1	32929863	32929862	**Bladder**; CRC; multiple myeloma	**BLCA**, LUSC
** **	22q13.32	22q13.32	22	48026910	51304566	3277656	**Endometrial**; Pancreatic	BLCA, GBM, LUAD, OV, **UCEC**

SNP associations and tumor types highlighted in bold indicate those whereby cancer type of SNP associations matches tumor type in which recurrent SCNAs were identified.

* Chromosomal locations based on human genome build hg19.

** Cancer type of SNP association loci that overlap SCNA regions (SNPs retrieved from January 2015 version of NHGRI GWAS catalog).

‡ Tumor type in which recurrent SCNAs were detected in TCGA.

ALL = acute lymphoblastic leukemia; BLCA = bladder; BRCA = breast; CLL = chronic lymphoblastic leukemia; CRC = colorectal; GBM = glioblastoma multiforme; HNSC = head and neck squamous cell carcinoma; KIRC = kidney renal cell carcinoma; LAML = acute myeloid leukemia; LUAD = lung adenocarcinoma; LUSC = lung squamous cell carcinoma; OV = serous ovarian carcinoma; UCEC = endometrial (uterine).

## Discussion

Our group and others have previously shown that heritable genetic variants can provide a substrate for selection during tumor evolution in cancer [2, 4–7, 9]. Here, we describe a novel methodology, SMART-ddPCR, which allows high sample throughput and accurate assessment of both PAI and the associated somatic copy number alterations in tumor DNA.

Droplet digital PCR enables absolute quantitation of DNA copy number [25] and thus provides a precise measure of allelic proportions in SNP heterozygotes. This allows assessment of the hypothesis that risk alleles of heritable cancer-associated SNPs will be preferentially retained/gained when somatic loss/gain occurs in the tumor. The accuracy of this assay was demonstrated by measurements of allelic copy number in constitutional DNA, which resulted in risk allele proportions at approximately the expected value of 0.5 for all assays tested. The low SD values calculated from repeat measurements of allelic copy number, and hence the extremely low standard error values for both constitutional and tumor DNA, confirmed the high precision of SMART-ddPCR.

Gene copy number estimates were also accurate, as shown by the high level of agreement between *CDKN2A* and *IKZF1* copy number measured by SMART-ddPCR and that measured by MLPA using an established childhood ALL-specific probeset [16]. Moreover, the risk allele proportions for SNPs on chromosomes gained in HeH ALL were clustered around the expected values for diploid to triploid/tetraploid shifts in chromosomal copy number. Previous technologies used for assessing tumor PAI, including Sanger sequencing [6], microsatellite genotyping [2, 5], SNP genotyping [7] and quantitative PCR [4], do not yield the accurate measures of DNA copy number that are possible with digital PCR. We have demonstrated the reduced accuracy and sensitivity of Sanger sequencing compared with SMART-ddPCR for AI detection for two of the SNPs in this study. Thus, although previous studies reported significant PAI in tumor DNA, they did not elucidate the underlying somatic alterations causing allelic imbalance, and they may have missed subtle differences in allelic proportions that would only be detectable via measurement of absolute copy number. A summary of the attributes of SMART-ddPCR compared with those of methods used in previous studies is presented in S5 Table.

There are some limitations to the SMART-ddPCR method, as performed on the BioRad QX100. Although the QX100 allows for high sample throughput of up to 96 samples per run, it is only optimized for assessment of a single SNP locus at a time. This is adequate for investigation of known cancer-associated SNPs that are candidates for tumor PAI, as described in this study, but is not optimal for identifying novel loci on a larger-scale. An additional problem that applies to all methods for PAI assessment, including this study, is that it is only possible with SNP heterozygotes, so large sample sizes are required to investigate SNPs with low minor allele frequencies. In this study, thresholds of AI were defined for each SNP using a stringent cut-off of three standard deviations (*i*.*e*. three-sigma rule) above or below the mean risk allele proportion measured in heterozygotes with available constitutional DNA. In studies where constitutional DNA is available for all subjects, it may be preferable to define AI based on paired tumor-normal comparisons for each subject, which could increase the power to detect significant tumor PAI. Finally, this study was carried out using diagnostic bone marrow DNA from ALL cases, in which the vast majority of cells are leukemic. To investigate PAI in solid tumors, the tumor purity will need to be considered.

In this study, we applied SMART-ddPCR to assess potential tumor PAI for childhood ALL-associated SNPs, as these are located either within genomic regions of somatic loss or on chromosomes commonly gained in the HeH subtype. We recently reported that the *CDKN2A* missense SNP rs3731249 shows tumor selection of the risk allele in heterozygote cases with somatic *CDKN2A* loss, with 14 patients with preferential retention of the risk allele versus only 3 with retention of the protective allele [9]. Here, though the *CDKN2A* tagging SNP rs3731217 did show a trend in the same direction, it did not show significant tumor PAI. This was not entirely unexpected, as rs3731217 was in weak linkage disequilibrium with the missense SNP in our data, and was less strongly associated with ALL risk [9]. Given the ratio of rs3731217 heterozygotes with AI favoring the risk allele (*n* = 11) compared with those favoring the protective allele (*n* = 6), we would have required at least double the number of samples (*i*.*e*. 24 vs. 13) to detect significant PAI (p<0.05) using a 1-tailed binomial significance test. Similarly, the *IKZF1* SNP rs4132601 showed a non-significant trend towards preferential retention of the risk allele and loss of the protective allele in the tumor, for which we would have required almost 4-fold the number of samples (61 vs. 43) to reveal significant PAI. This SNP maps to the 3’ end of *IKZF1* and does not appear to have any functional consequences [11]. Furthermore, the effect size of rs4132601 on ALL risk is ~1.5 to 1.7 [11, 19], hence there are likely to be as yet unidentified functional variants underlying the *IKZF1* association signal that may show significant tumor PAI if tested. This highlights the need to advance studies on identification of functional alleles (rather than GWAS “tag” SNPs) prior to assessments of PAI.

The ALL-associated SNP in *GATA3* did not show tumor PAI in DNA from HeH subjects heterozygous for the risk allele, which was predicted given that this locus is exclusively associated with the Ph-like subtype, and not HeH ALL [24]. For the HeH-associated SNPs in *CEBPE*, *ARID5B*, and *PIP4K2A*, we did not see significant preferential gain of risk alleles in tumor samples from heterozygous patients. This may be due to hyperdiploidy being a primary leukemogenic event that likely arises from a single abnormal mitosis [32, 33]. Hence, there is a 50:50 chance of a risk allele being amplified, after which there are no additional chromosomal gains that would provide a substrate for tumor selection. Therefore, the selective pressure during the initial hyperdiploid event would be less than that for SNPs within regions of secondary tumor alterations, such as deletions of *CDKN2A* and *IKZF1*.

Indeed, we found a surprisingly complex assortment of somatic alterations at these two loci, in particular for *CDKN2A*, where we detected apparent homozygous and hemizygous deletions, as well as copy-neutral LOH with varying degrees of clonality. Recent studies of clonal evolution in childhood ALL also showed that *CDKN2A* and *IKZF1* deletions are secondary leukemogenic events that can be subclonal [32, 34–36]. However, this is the first report of copy-neutral LOH at the *IKZF1* locus in ALL, which we might expect to occur given the frequency of *IKZF1* gene deletions, which in turn create an opportunity for gene conversion leading to LOH. Deletion of *IKZF1* is associated with poor outcome in childhood ALL [37], and it is possible that copy-neutral LOH at the *IKZF1* locus may also affect outcome if the resulting homozygosity includes SNP risk alleles associated with reduced *IKZF1* expression, such as for rs4132601 [11]. In contrast to *CDKN2A*, we did not detect any homozygous *IKZF1* deletions. Previous studies have demonstrated that homozygous loss of *IKZF1* is extremely rare in ALL cases [15, 16, 37], suggesting that loss of only one functional copy of this gene has strong leukemogenic effects. This may also explain the lack of significant tumor PAI for the *IKZF1* SNP rs4132601, as hemizygous *IKZF1* loss has such a strong leukemogenic effect that there is less selective pressure on the remaining allele. Alternatively, an obligatory functional requirement for a small level of IKZF1 may exist for the leukemic cell.

Childhood ALL-associated SNPs, located in genomic regions of somatic loss or gain, provided ideal candidate loci for development of our novel methodology for tumor PAI assessment. SMART-ddPCR is a useful tool to investigate any heritable cancer-associated SNPs located within regions of known somatic alterations, therefore we wished to determine potential candidates for tumor PAI across different cancer types. By analyzing the overlap of recurrent SCNAs from TCGA [29] and cancer-associated SNPs from the NHGRI GWAS Catalog [30], we discovered that over 10% (16/140) of recurrent SCNA loci overlap with heritable variants associated with a matching cancer type and thus may provide a substrate for selection during tumor evolution. Obvious candidates for investigation are *TERT* at 5p15.33 and *MYC* at 8q24.21, in which SCNA were identified in multiple cancer types matching SNP associations in those genomic regions. Indeed, previous studies have found evidence of tumor PAI for SNPs at 8q24.21 in colorectal cancer [6, 38]. Furthermore, a previous study found evidence of tumor PAI at the *EGFR* locus, one of our candidate amplification loci, through *in silico* analysis of glioblastoma data from TCGA, with preferential amplification of a SNP allele (rs13222385, G) that was associated with increased EGFR expression [39]. It should be noted that the TCGA dataset was limited to 11 cancer types, and only includes recurrent SCNAs that appeared in more than one tumor type [29]. SNPs included in our analysis were identified by GWAS, and do not include associations discovered through candidate gene analyses. Therefore, there are likely additional genomic regions containing heritable SNPs that may undergo selection during tumor evolution.

In conclusion, SMART-ddPCR is a highly accurate method for assessing tumor PAI of heritable genetic variants and elucidating the somatic alterations underlying allelic imbalance, as well as providing information on the clonality of such alterations. While PAI was evident at some childhood lymphocytic leukemia loci, it was not a universal feature of this disease. This methodology is pertinent to all cancer types, in particular those in which known recurrent SCNAs overlap heritable SNPs associated with the same type of cancer.

## Supporting Information

S1 Fig Sanger sequencing assessment of AI for *IKZF1* SNP rs4132601. PCR and sequencing was carried out for 6 constitutional DNA samples and 13 tumor DNA samples from ALL patients heterozygous for rs4132601. A: Mean risk allele proportions displayed as a fraction of the total amount of both alleles based on measures of chromatogram peak heights, and across forward and reverse sequencing data (error bars represent the standard error of the mean from these “repeat” measures). Upper/lower thresholds of AI were +/- 3 SDs from the mean allelic proportion from repeat measurements in constitutional DNA samples (white squares). Red horizontal bars represent risk allele proportions for the same samples as measured by SMART-ddPCR. B: Comparison between risk allele proportions measured by Sanger sequencing and by SMART-ddPCR, showing high correlation. The circled sample was determined to have AI favoring the protective allele by SMART-ddPCR but was not shown to have AI by Sanger sequencing. C: Examples of sequence graphs showing relative peak heights for the risk allele (G) and protective allele (T) in constitutional DNA, in a sample with AI favoring the risk allele, and in a sample with AI favoring the protective allele.
(TIF)

S2 Fig Sanger sequencing assessment of AI for *ARID5B* SNP rs7089424. PCR and sequencing was carried out for 7 constitutional DNA samples and 17 tumor DNA samples from ALL patients heterozygous for rs7089424. A: Mean risk allele proportions displayed as a fraction of the total amount of both alleles based on measures of chromatogram peak heights, and across forward and reverse sequencing data (error bars represent the standard error of the mean from these “repeat” measures). Upper/lower thresholds of AI were +/- 3 SDs from the mean allelic proportion from repeat measurements in constitutional DNA samples (white squares). Red horizontal bars represent risk allele proportions for the same samples as measured by SMART-ddPCR. B: Comparison between risk allele proportions measured by Sanger sequencing and by SMART-ddPCR, showing high correlation. The circled sample was determined to have AI favoring the protective allele by SMART-ddPCR but was not shown to have AI by Sanger sequencing. C: Examples of sequence graphs showing relative peak heights for the risk allele (G) and protective allele (T) in constitutional DNA, in a sample with AI favoring the risk allele, and in a sample with AI favoring the protective allele.
(TIF)

S1 Table Risk allele proportion measurements in constitutional DNA from SNP heterozygotes. (XLSX)

S2 Table Recurrent somatic amplification loci in TCGA and information on overlapping cancer-associated GWAS SNPs. (XLSX)

S3 Table Recurrent somatic deletion loci in TCGA and information on overlapping cancer-associated GWAS SNPs. (XLSX)

S4 Table Cancer-associated NHGRI GWAS SNPs overlapping the TCGA recurrent SCNA loci. (XLSX)

S5 Table Comparison of the attributes of SMART-ddPCR with those of other methods used in previous studies of tumor PAI. (XLSX)

S6 Table Raw data from ddPCR experiments for each of the SNPs tested in this study. (XLSX)
